# Urban–suburban differences in the response of typical greening tree species to extreme drought in a subtropical city

**DOI:** 10.3389/fpls.2026.1900663

**Published:** 2026-08-10

**Authors:** Youmei Lei, Xinyu Ren, Junfei Lin, Xiaofeng Liao, Siyi Yang, Xing Pu

**Affiliations:** 1 Key Laboratory of Southwest China Wildlife Resources Conservation (China West Normal University), Ministry of Education, Nanchong, China; 2 College of Life Science, China West Normal University, Nanchong, China

**Keywords:** drought stress, ecological resilience, subtropical urban forest, tree-ring width, urban heat island

## Abstract

**Introduction:**

Against the backdrop of global change, the mechanisms by which the urban heat island effect and climate warming influence tree growth in urban versus suburban areas remain poorly understood.

**Methods:**

We collected tree core samples of four common greening tree species from urban and suburban areas of Nanchong, a subtropical city in Sichuan Province, and established tree-ring width chronologies to analyze differences in radial growth responses to climatic factors.

**Results:**

Over the past decade, the growth rate of suburban trees was significantly higher than that of urban trees. Water availability was the key factor limiting urban tree growth, whereas rising temperatures promoted tree growth in both urban and suburban areas to a certain extent. Suburban trees were more sensitive to spring temperature, while summer high temperature had a more pronounced effect on urban trees. During extreme drought events, suburban trees exhibited higher recovery and resilience, likely attributable to the more favorable water environment in suburban areas. Ring-porous poplar exhibited the lowest resistance and resilience during extreme droughts, whereas the diffuse-porous evergreen broadleaf *Cinnamomum camphora* maintained relatively high recovery in urban areas, suggesting a native species advantage for drought buffering.

**Discussion:**

This study reveals the urban–suburban differentiation in tree growth responses to climate change under urbanization, providing a theoretical basis for enhancing the climate adaptability and scientific management of urban forests in subtropical cities.

## 1 Introduction

Global climate change is reshaping terrestrial ecosystems at an unprecedented rate (Gu et al., 2025; Reek et al., 2026). The IPCC Sixth Assessment Report (IPCC, 2023) clearly indicates that since the mid-20th century, the frequency of extreme heatwaves and compound drought events has significantly increased, particularly in the East Asian monsoon region. Southwest China, characterized by a typical subtropical humid monsoon climate, has experienced frequent high intensity, long duration regional extreme droughts in recent years (e.g., the 2006 Sichuan Chongqing drought), posing serious challenges to regional ecological security (Yan Y. et al., 2024; Yu et al., 2023; Zhang, X., et al., 2024). At the same time, rapid urbanization intensifies local environmental disturbances, creating a pronounced urban heat island (UHI) effect, where urban temperatures are generally 1–4 °C higher than those in surrounding rural areas (Esperon-Rodriguez et al., 2022; Guo et al., 2026; Schneider et al., 2022). This is accompanied by multiple stresses such as soil compaction, restricted water infiltration, and enhanced evapotranspiration, further compressing the living space of urban trees (Esperon-Rodriguez et al., 2025a; Stefanidis et al., 2023; Zhang et al., 2022).

In this context, urban forests serve as critical green infrastructure for regulating microclimate, sequestering carbon, and improving human well-being. The sustainability of their ecological functions depends heavily on the adaptive capacity of trees to climate change, i.e., “ecological resilience”-the ability of an ecosystem to maintain its structure and function after a disturbance (Lloret et al., 2011). However, existing studies indicate that the growth dynamics of urban trees are closely related to their local environment, and the response patterns of urban versus suburban trees to climatic factors may differ systematically (Nitschke et al., 2017). Although traditional ecological monitoring approaches (e.g., remote sensing, physiological measurements) can provide short term response information, they are limited in revealing long term, continuous growth histories and their intrinsic links to climate variability. In contrast, dendrochronology, with its high temporal resolution, precise dating, and high sensitivity to environmental changes, has become a powerful tool for disentangling tree climate relationships and quantifying ecological resilience (Dong and Fang, 2024; Esperon-Rodriguez et al., 2025b; Wang et al., 2025; Wu et al., 2025).

In recent years, dendrochronological methods have been gradually introduced into urban ecological research (Bernal-Escobar et al., 2025; Wu et al., 2025; Zhang, Q., et al., 2024). Internationally, A previous study found in southeastern Australia that urban eucalypts and ashes exhibited significantly higher sensitivity to summer drought than their suburban conspecifics, with stronger correlations between ring width and the Standardized Precipitation Evapotranspiration Index (SPEI) (Nitschke et al., 2017). A study further confirmed that although the UHI effect may extend the growing season of some tree species, it also exacerbates water stress, leading to greater growth suppression in urban trees during extreme drought years (Rochner et al., 2025). A global meta-analysis reported that about 60% of urban tree species have shown an increasing sensitivity to drought, suggesting a decline in their climate resilience (Esperon-Rodriguez et al., 2022). Recent studies across multiple cities have revealed that the existence of an urban tree resilience gradient, characterized by nonlinear declines in both resistance and recovery along the urbanization intensity gradient (Esperon-Rodriguez et al., 2025c; Wu et al., 2025; Yang et al., 2026). Several studies have begun to operationalize the concept of “ecological resilience” into quantifiable tree ring metrics (Esperon-Rodriguez et al., 2025b; Yang et al., 2026). This framework has been successfully applied to trees in Mediterranean (Gazol et al., 2017), temperate (Schneider et al., 2022), and even tropical cities (Kabano et al., 2021; Moser-Reischl et al., 2018). Although urban trees had high growth rates, their resilience indicators were significantly lower than those of suburban individuals, indicating that “high growth does not mean high resilience” (Franceschi et al., 2023). Similarly, the urban tree resilience is driven not only by climate but also strongly depends on soil water availability and root space in Louisville, Kentucky (Rochner et al., 2025).

However, existing urban dendrochronological studies have focused mainly on temperate or Mediterranean climates (e.g., Europe, North America, Australia), while research in the subtropical humid monsoon region of China remains extremely scarce (Wu et al., 2025; Zhang et al., 2024). This region is characterized by “concurrent rain and heat, distinct dry and wet seasons”, and rapid urban expansion, but whether native greening tree species (e.g., *Cinnamomum camphora*, *Metasequoia glyptostroboides*) possess sufficient climate adaptation potential remains unclear. Moreover, the responses of different tree species to drought may differ fundamentally (Schneider et al., 2022; Warner et al., 2024; Yang et al., 2026). For example, ring porous species (e.g., poplar) have large earlywood vessels and are prone to embolism under drought, leading to impaired water transport, whereas diffuse porous or coniferous species usually have higher embolism resistance (Choat et al., 2012; Li et al., 2026). Therefore, if urban greening practices ignore interspecific differences in response and blindly introduce highly sensitive exotic species, large scale tree decline or mortality may occur under future extreme climate events (Esperon-Rodriguez et al., 2025b, Esperon-Rodriguez et al., 2025c; Wood and Dupras, 2021).

How the UHI effect and climate warming affect tree growth across urban–suburban gradients and among different species is still unresolved, especially concerning the influence of extreme drought events. The present study takes Nanchong City, Sichuan Province, as a typical case. Four widely planted representative greening tree species were selected: *Metasequoia glyptostroboides*, *Cinnamomum camphora*, *Populus* ‘*Zhonghua Hongye*’, and *Cedrus deodara*. By establishing high precision tree ring chronologies, we systematically compare their growth responses to climate change (especially extreme drought events) between urban and suburban areas, and quantify their ecological resilience using the framework of Lloret et al. (2011) in a subtropical urban context. We propose the following scientific hypotheses: H1: Under the dual stresses of UHI effect and soil water limitation, urban trees show significantly higher sensitivity to extreme drought events than their suburban conspecifics, manifested as lower resistance and recovery. H2: Different functional types exhibit distinct drought responses; ring porous species (poplar) are more vulnerable to short term water stress and have lower resilience than diffuse porous (camphor) or coniferous species (cedar, dawn redwood). H3: As urbanization intensifies, the radial growth–climate correlation of urban trees will strengthen over time, reflecting a stress accumulation effect and leading to a continuous decline in resilience. To test these hypotheses, we used dendrochronological methods and established paired plots in the main urban area (parks and street green spaces in built up areas) and surrounding rural areas (farmland forest ecotones) of Nanchong, ensuring consistent climatic background but a clear anthropogenic disturbance gradient. The objectives of this study are: (1) to reveal the differences in growth responses of urban versus suburban trees to climate change, especially extreme drought; and (2) to quantify the ecological resilience (resistance, recovery, and resilience) of common greening tree species (camphor, poplar, cedar, dawn redwood) and their urban suburban differentiation patterns. The results provide empirical evidence and management recommendations for scientifically selecting drought tolerant tree species and enhancing the climate adaptability of urban forests in subtropical cities.

## 2 Materials and methods

### 2.1 Study area

The study was conducted in Nanchong City (106°E, 30.8°N), Sichuan Province, China. Nanchong has a typical subtropical humid monsoon climate, with a mean annual temperature of 17.1 °C and mean annual precipitation of 1020 mm. Precipitation is concentrated from May to September, with distinct dry and wet seasons. In the past two decades, under the influence of climate change, extreme high temperature and drought events have occurred frequently, including regional severe droughts in 2006 and 2011. The built-up area of the city is approximately 180 km^2^, and the UHI intensity (urban suburban temperature difference) can reach 2.5–3.8 °C in summer (Yan et al., 2026).

### 2.2 Plot establishment and core collection

**Figure 1:**
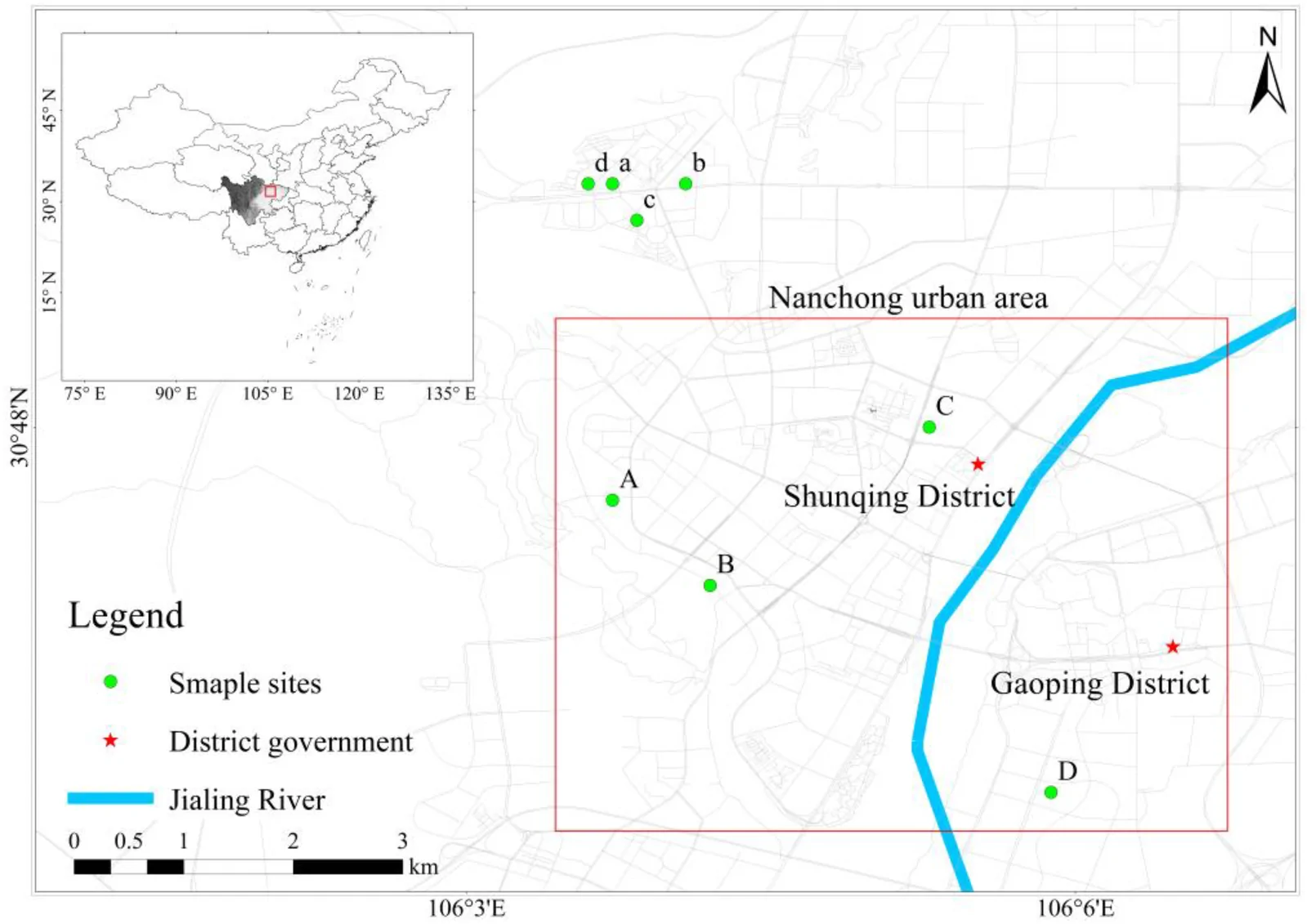
Geographical distribution of tree-ring sampling sites in urban and suburban areas of Nanchong, a subtropical city in Southwest China. **(A–D)** represent urban *Metasequoia glyptostroboides*, *Cedrus deodara*, *Cinnamomum camphora*, and *Populus‘Zhonghua Hongye’*, respectively; lowercase letters represent the corresponding suburban trees.

Eight paired plots were established in the main urban area (high-density built-up area: parks and street green spaces) and eight in the surrounding rural area (mainly farmland forest mosaics) during 2021 to 2023 (**Figure 1**). The target tree species were four widely planted greening species: *Metasequoia glyptostroboides* (dawn redwood), *Cinnamomum camphora* (camphor), *Populus* ‘*Zhonghua Hongye*’ (poplar), and *Cedrus deodara* (cedar). For each species, ≥10 healthy adult individuals (free from obvious pests, diseases, or mechanical damage) were selected in both urban and suburban areas. Tree cores were extracted at breast height (1.3 m) using a 5.15 mm increment borer. The cores were placed in plastic straws, labeled, and stored in the dark under dry conditions. A total of 179 cores were collected (**Supplementary Table 1**).

### 2.3 Ring processing and chronology development

All cores were progressively sanded with sandpaper (grit size from 120 to 800 mesh) until ring boundaries were clearly visible. Ring widths were measured to the nearest 0.001 mm using the WinDENDRO tree ring analysis system (Regent Instruments, Canada), and preliminary cross dating was performed. Cross dating quality was further verified using the COFECHA program. Detrending was performed using negative exponential curves or linear regression (Cook, 1985) in the ARSTAN program. Standard (STD) chronologies were developed for each species in urban and suburban areas for subsequent climate response analysis. This classic approach was selected because it effectively models biological age-related growth trends while preserving high-frequency climate signals relevant to our research objectives (Fritts, 1976). Although cubic smoothing splines offer greater flexibility, they carry a risk of overfitting and potentially removing climatic information. Given the moderate length of our series and our sampling design averaging multiple individuals per site, this approach is appropriate and robust for our study. For growth trend analysis, ring widths were converted to basal area increment (BAI, cm^2^·yr^-^¹) using the formula (Phipps and Whiton, 1988):


$$BAI=\pi \times \left(R_{n}^{2}-R_{n-1}^{2}\right)$$

where R is the tree radius and n is the year of ring formation. Raw BAI series were used to assess long term growth trends because BAI is less affected by biological variation than ring width and more directly reflects stem biomass. BAI calculations was performed using the R package ‘*dplR*’ (Bunn, 2008).

For growth trend analysis, we followed protocols established in earlier work (Gaire et al., 2023; Wu et al., 2025). Generalized additive mixed models (GAMMs) were applied to remove age- and size-dependent growth trends from the BAI series (Wood, 2017). To improve distribution normality, BAI values were log-transformed. The GAMM specification was as follows:


log(BAI)~log(BA)+s(Age)+(1|TreeID)+corAR1

Here, BAI (basal area increment, cm^2^·yr^-^¹) served as the response variable, while BA (basal area, mm^2^) and Age (tree age in years) were predictors. TreeID was included as a random effect for each individual tree. A continuous first-order autoregressive structure (corAR1) was incorporated, with year as a temporal covariate and TreeID as a grouping factor, to account for temporal autocorrelation (Gaire et al., 2023). Using the ‘*mgcv*’ package (Wood, 2017), we modeled BAI residuals to examine growth rate trends for each tree species at each sampling site. BAI residuals were calculated as:


$$BAI_{residuals}\sim log\left(BAI_{observed}\right)-log\left(BAI_{predicted}\right)$$

Linear trends in BAI residuals at each site were evaluated using linear mixed-effects models (LME) from the ‘*nlme*’ package in R (Pinheiro et al., 2021):


$$BAI_{residuals}\sim Year+\left(1|TreeID\right)+corAR1$$

where BAIresiduals represents the GAMM-derived basal area increment residuals, and TreeID is a random factor for individual trees.

To test for differences in growth trajectories between urban and suburban trees, we employed a linear mixed-effects model (LME) examining the interaction between calendar year and site (Gaire et al., 2023):


$$BAI_{residuals}\sim Year\times Site+\left(1|TreeID\right)+corAR1$$

In this model, Year and Site were treated as fixed factors, and their interaction term (Year×Site) was also considered a fixed effect (Gaire et al., 2023). To identify shifts in growth trends, we used the ‘*segmented*’ R package (Muggeo, 2017) to detect breakpoints in the residuals of individual BAI series. By analyzing the density distribution of these breakpoints, we pinpointed the year at which the majority of trees exhibited a mean breakpoint frequency (Gaire et al., 2023). Consequently, the BAI residuals were split into two sub-periods to enable a more detailed analysis of growth trends.

### 2.4 Climate data and identification of extreme drought events

**Figure 2:**
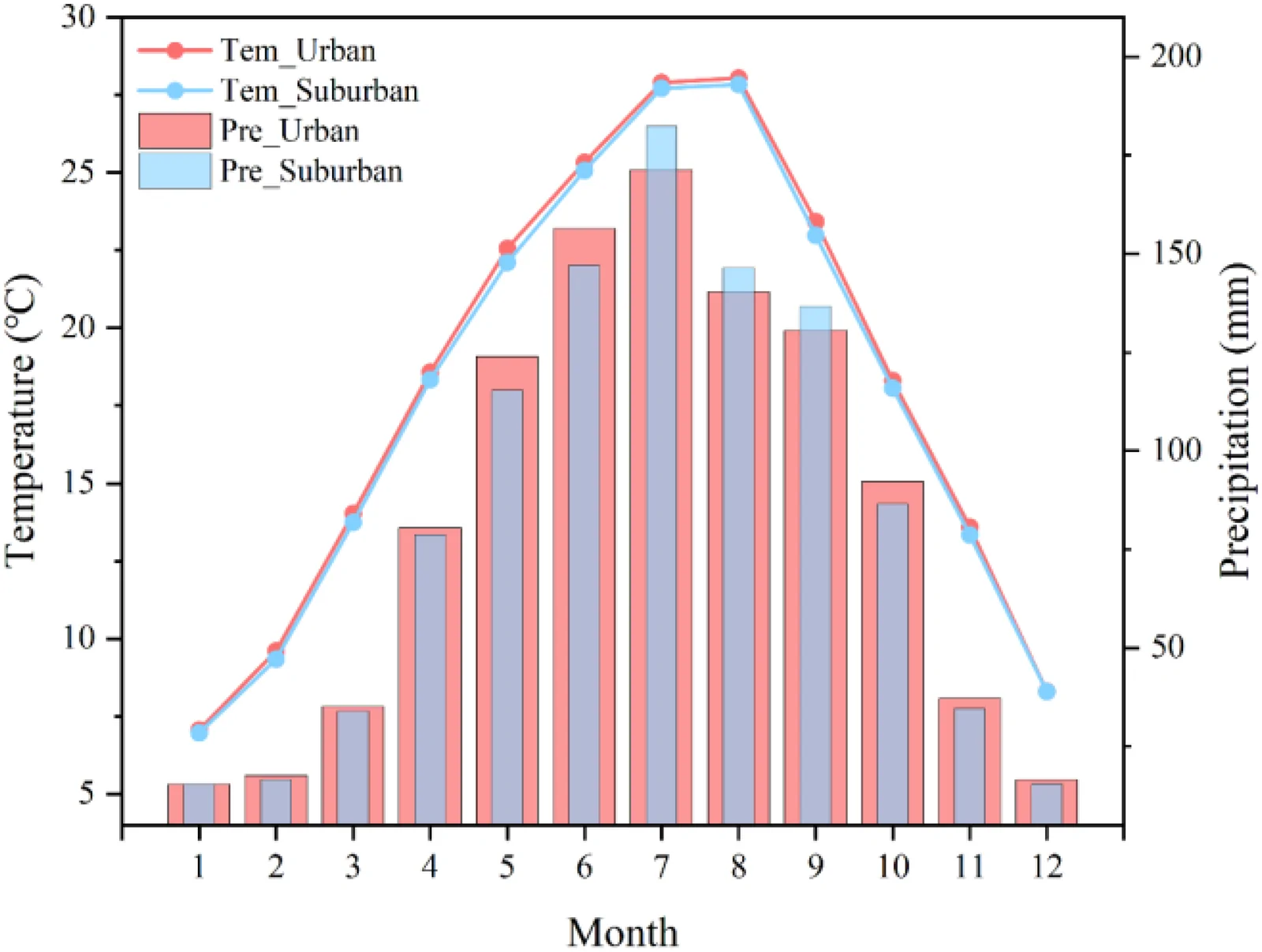
Monthly average precipitation (bar) and temperature (line) of the studied urban and suburban areas from 1995 to 2022 in Nanchong, China.

Monthly mean precipitation and temperature for urban and suburban areas were obtained from the Gaoping meteorological station (30°47′N, 106°06′E, 347 m a.s.l.) and the Xichong meteorological station (30°58′N, 105°52′E, 361.2 m a.s.l.), respectively, via the China Meteorological Data Service Centre (https://data.cma.cn/). The Gaoping meteorological station located within the Nanchong urban built-up area, and the Xichong meteorological station located in a suburban agricultural region approximately 20–25 km from the city center, were selected to represent urban and suburban climatic conditions, respectively. Both stations are national basic stations with long-term quality-controlled records. The minimal elevation difference (~14 m) ensures that the observed urban–suburban temperature differences primarily reflect urbanization effects rather than altitudinal gradients. To further analyze the impact of drought on tree radial growth in the two areas, the Standardized Precipitation Evapotranspiration Index (SPEI) was downloaded from the KNMI Climate Explorer (https://climexp.knmi.nl) for the grid point closest to the sampling sites (0.5° × 0.5° resolution) for the period 1995–2022. The Standardized Precipitation Evapotranspiration Index (SPEI) at the 6-month timescale (SPEI-6) was used for drought characterization. This timescale was selected after preliminary evaluation of 1-, 3-, 6-, and 12-month scales, as it best captures cumulative seasonal water deficits relevant to tree radial growth in this subtropical humid region and corresponds temporally to the medium-term ecological dynamics represented by the resilience indices. Based on the criterion SPEI< −1, typical extreme drought events were identified: 2006 and 2011. These events were highly consistent with historical disaster records and were selected as the time windows for resilience analysis. From 1995 to 2022, the temperature and precipitation changes in the urban and suburban areas of Nanchong showed that the urban areas became warmer and drier (**Figure 2**; **Supplementary Figure 1**). The annual mean temperature and total precipitation in the region both showed significant increasing trends, while the SPEI trend was not significant (**Supplementary Figure 1**).

### 2.5 Calculation of ecological resilience indicators

Following the formulas proposed by Lloret et al. (2011), resistance (Rt), recovery (Rc), and resilience (Rs) were quantified for each stress event:


Rt=Dr/PreDr

Rc=PostDr/Dr

Rs=PostDr/PreDr

where Dr is the radial growth in the year of the extreme drought event. To account for the “legacy effect” after extreme climate stress and the influence of adjacent stress events, the reference periods before and after the event were set to four years. PreDr and PostDr are the mean radial growth of the four years before and after the stress event, respectively. The study utilized the ‘*pointRes*’ package in R for analysis (van der Maaten-Theunissen et al., 2015).

### 2.6 Statistical analyses

Climate response analyses were performed using DENDROCLIM 2002 to calculate Pearson correlation coefficients between ring width indices and monthly climatic factors (temperature, precipitation, SPEI) from September of the previous year to October of the current year, generating response function plots. Multivariate regression models were used to examine the relationships between influencing factors and spatiotemporal patterns of tree growth in urban and suburban areas. Because the magnitudes of the independent variables differed, all independent variables were standardized before model construction. A stepwise backward regression approach was used to select the optimal model based on the Akaike information criterion (AIC). All analyses were carried out in R 4.3.2 (R Core Team, 2022).

## 3 Results

### 3.1 Trends in tree radial growth

**Figure 3:**
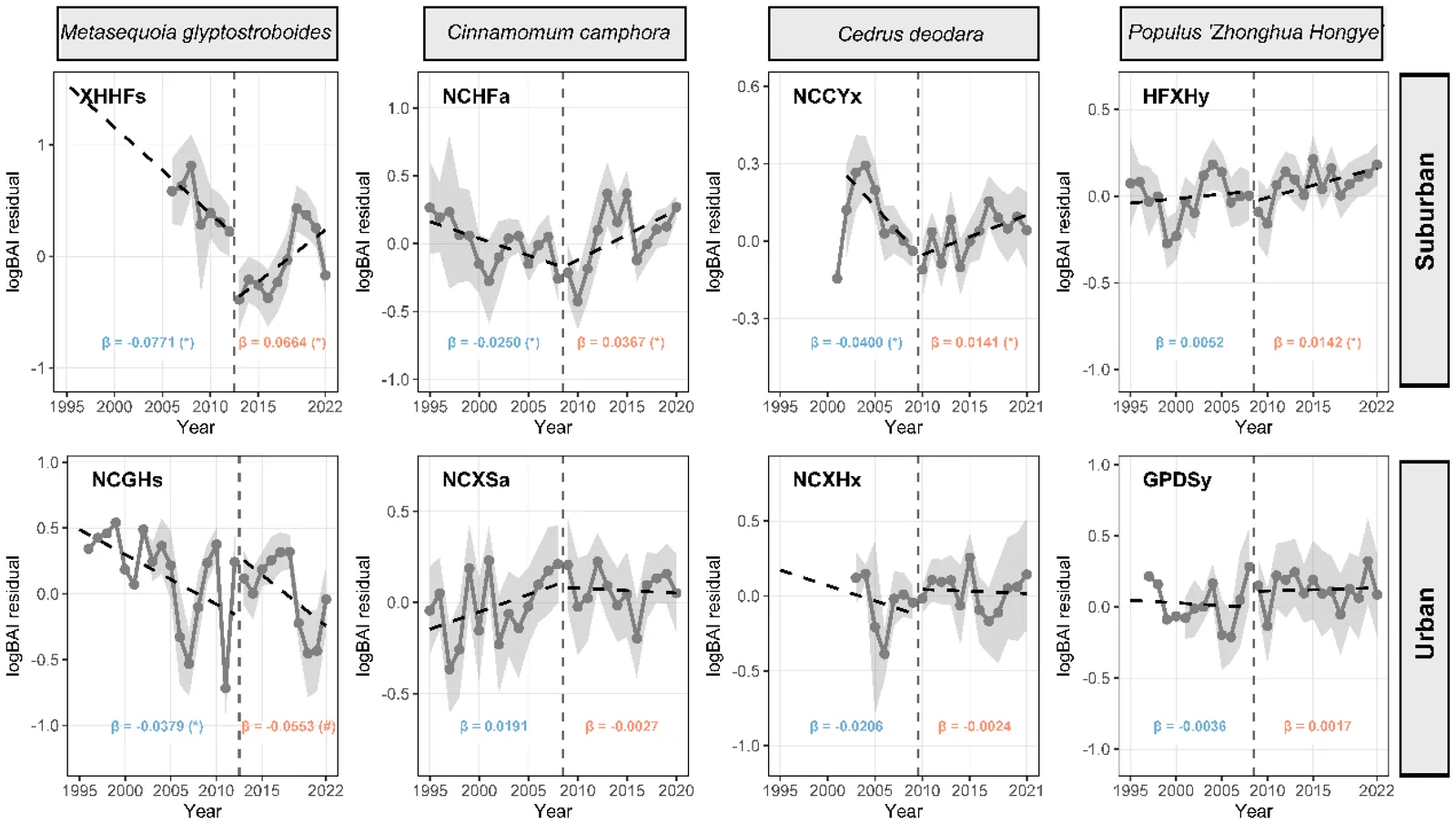
Growth trends (log BAI residuals) of urban and suburban trees for the common period 1995–2022. Gray-shaded areas indicate 95% confidence intervals. Asterisks represent significant correlations (#: *p<* 0.1, *: *p* < 0.05).

Growth trends for the four tree species were detected by fitting linear mixed-effects models to the BAI residuals obtained after detrending with GAMM (**Figure 3**; **Supplementary Figure 4**). Segmented regression analysis identified breakpoints in growth trajectories at 2012 for *M. glyptostroboides*, 2008 for *C. camphora* and *P. ‘Zhonghua Hongye’*, and 2009 for C. deodara. For suburban trees, growth trends shifted from negative or non-significant before the breakpoint to significantly positive afterwards (*M. glyptostroboides*: β = −0.0771, *p* < 0.05 to β = 0.0664, *p* < 0.05; *C. camphora*: β = −0.0250, *p* < 0.05 to β = 0.0367, *p* < 0.05; *C. deodara*: β = −0.0400, *p* < 0.05 to β = 0.0141, *p* < 0.05; *P. ‘Zhonghua Hongye’*: β = 0.0052, *p* > 0.05 to β = 0.0142, *p* < 0.05) (**Figure 3**). In contrast, urban trees showed non-significant or declining trends after the breakpoint, with the exception of *M. glyptostroboides* which exhibited a weakly positive trend (β = 0.0553, *p* < 0.1) (**Figure 3**). This indicates that the growth trend of suburban trees has shifted from a past decline to a recent increase, whereas urban trees show a declining or stagnant state. Based on the characteristic values of tree-ring chronologies, there are obviously differences between the trees in the urban area and those in the suburban area. (**Supplementary Table 2**). The growth decline rate of trees in urban areas has generally been higher than that in suburban areas during the past decade. The average annual growth decline rate of urban trees is between 0.23 and 0.30 mm/year, while that of suburban cedars and poplars is between 0.16 and 0.25 mm/year. The first-order autocorrelation coefficient (AC1) of suburban trees is generally higher than that of urban trees, indicating that the climate of the previous year has a greater potential impact on the radial growth of suburban trees in the current year. The mean sensitivity (MS) value of urban trees is relatively higher, suggesting that the urban trees may be highly sensitive to climatic and environmental factors. The expressed population signal (EPS) of all tree-ring series has reached the critical threshold of 0.85, indicating their high reliability and applicability for fitting analysis with climatic factors. The moderate average inter-series correlation (Rbar) values indicates that the inter-annual growth changes of trees within the study site are synchronous (**Supplementary Table 2**).

### 3.2 Tree growth–climate relationships

**Figure 4:**
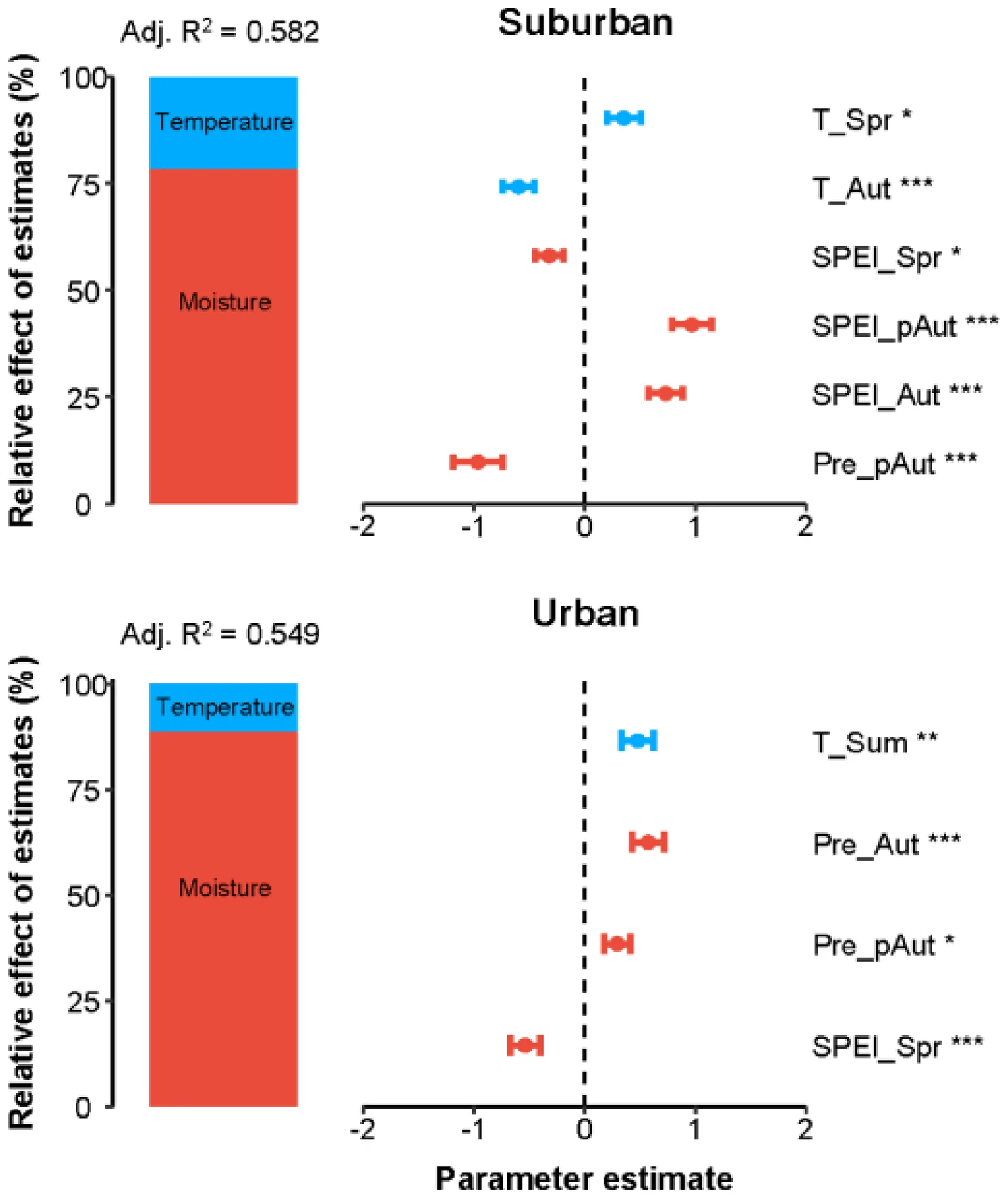
Effects of multiple environmental factors on urban and suburban trees growth. The symbols *, **, and *** denote significance levels at *p<* 0.05, *p<* 0.01 and *p<* 0.001, respectively. T_Spr = spring mean temperature; T_Sum = summer mean temperature; T_Aut = autumn mean temperature; SPEI_pAut = previous autumn standardized precipitation-evapotranspiration index; SPEI_Spr = spring standardized precipitation-evapotranspiration index; SPEI_Aut = autumn standardized precipitation-evapotranspiration index; Pre_pAut = previous autumn total precipitation; Pre_Aut = autumn total precipitation.

Correlation analyses between residual chronologies and climatic factors revealed differences in climate responses between urban and suburban trees (**Figure S3**). Stepwise regression models showed that water availability during the growing season is the main limiting factor for tree growth in both urban and suburban areas, with urban trees being more strongly affected by water availability (**Figure 4**). Spring and autumn temperatures significantly influenced suburban tree growth, while summer temperatures significantly affected urban tree growth (**Figure 4**). Suburban tree growth was mainly influenced by autumn SPEI (**Figure 4**). In contrast, urban tree growth showed a positive correlation with precipitation (**Figure 4**). The seasonal climate responses confirm that water availability is the primary factor limiting radial growth of trees in both urban and suburban areas.

### 3.3 Comparison of ecological resilience indicators

**Figure 5:**
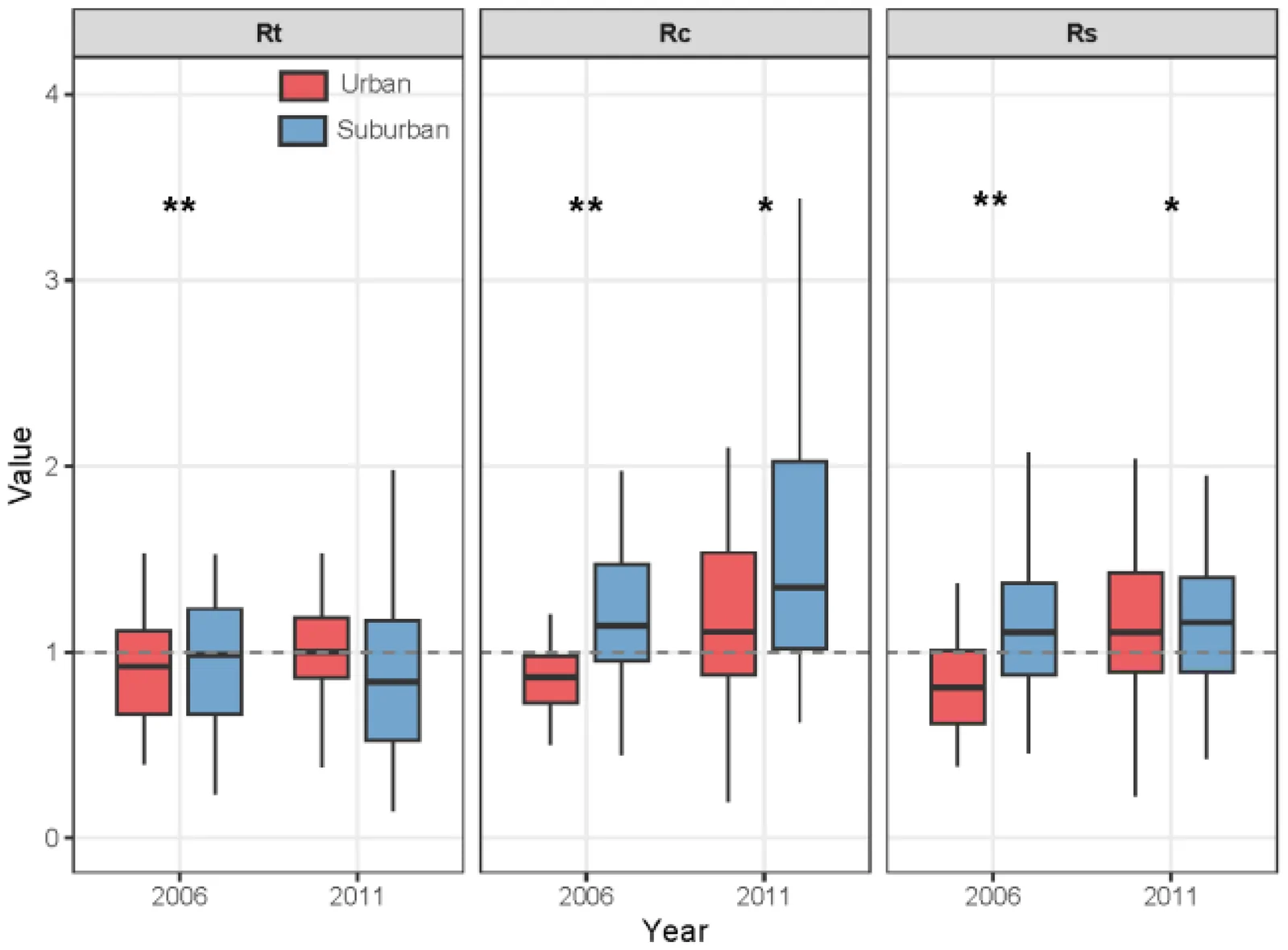
Comparison of resistance, recovery, and resilience between urban and suburban trees (*: *p* < 0.5, **: *p* < 0.01).

**Figure 6:**
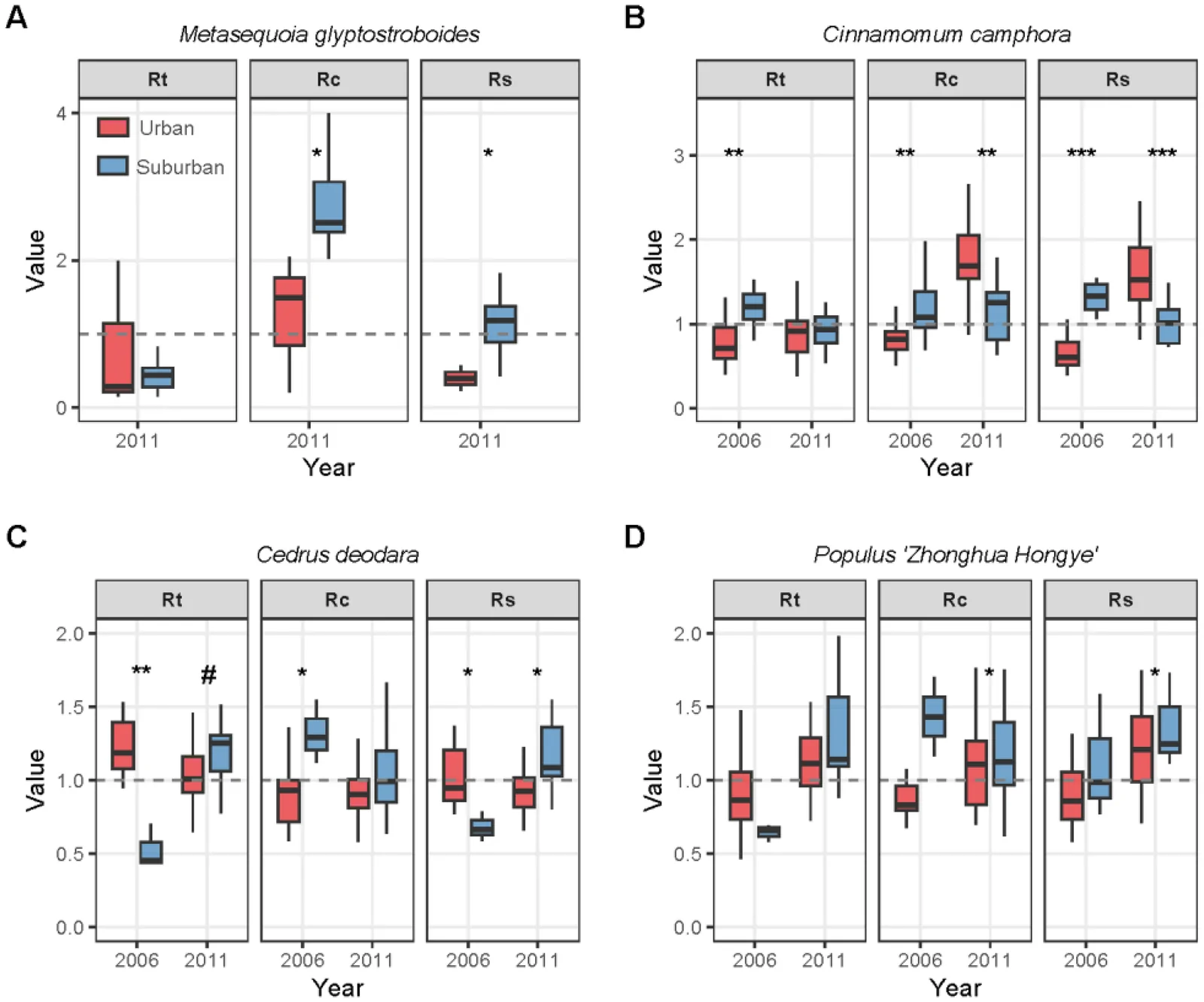
Comparison of resistance, recovery, and resilience among different tree species in urban and suburban areas (#: *p* < 0.1, *: *p* < 0.5, **: *p* < 0.01, ***: *p* < 0.001).

Resilience indicator analysis showed that the recovery (Rc) and resilience (Rs) of suburban trees were significantly higher than those of urban trees (*p* < 0.05) (**Figure 5**). Among all species, suburban *M. glyptostroboides* had the highest recovery, while urban *C. deodara* had the lowest recovery; suburban poplar had the highest resilience, while urban *M. glyptostroboides* had the lowest resilience; urban *C. deodara* had the highest resistance, and urban *M. glyptostroboides* had the lowest resistance. Resistance (Rt) patterns differed between the two drought events: in 2006, suburban trees overall showed significantly higher Rt than urban trees (*p* < 0.05), with the exception of *Cedrus deodara* (urban > suburban, *p* < 0.01); in 2011, no significant overall difference was detected (**Figure 5**). During the 2011 drought, the resilience and recovery of urban *C. camphora* were significantly higher than those of suburban *C. camphora* (**Figure 6**).

## 4 Discussion

### 4.1 Tree radial growth responses to urbanization and climate change

Our study found that under the background of recent climate warming, the radial growth rate of urban trees in Nanchong City was lower than that of suburban trees (**Figure 3**; **Supplementary Figure 4**). Urban trees exhibited a declining or fluctuating trend, while suburban trees showed an increasing trend, which is consistent with most previous studies (Franceschi et al., 2023; Moser-Reischl et al., 2018; Nitschke et al., 2017). However, some studies have reported that urban warming can promote tree growth (Dahlhausen et al., 2018; Esperon-Rodriguez et al., 2025b; Zhang, Q., et al., 2024). This discrepancy may arise from differences in regional climate, tree species characteristics, and urban management practices (e.g., irrigation, pollution) (Dahlhausen et al., 2018; Warner et al., 2024; Yang et al., 2026). Urban environments are typically characterized by high runoff, low infiltration, and soil compaction, which further exacerbate water limitation (Esperon-Rodriguez et al., 2025c; Percival, 2023).

In this study, the key climatic factor limiting tree growth in both urban and suburban areas was not temperature but water stress. Although urban temperatures were higher than suburban temperatures (**Figure 2**; **Supplementary Figure 1**), radial growth was primarily regulated by water availability (**Figure 4**). The reduced sensitivity of tree growth to temperature has been documented in many forest sites worldwide (Keyimu et al., 2021; Roetzer et al., 2021; Wu et al., 2025). The physiological mechanism is as follows: rising temperatures increase transpiration rates and vapor pressure deficit, inducing stomatal closure and limiting CO_2_ uptake and carbon assimilation. At the same time, enhanced soil evaporation reduces water availability, inhibiting root uptake and cambial activity, ultimately limiting carbon reserves and growth (Chaudhry and Sidhu, 2022; McDowell et al., 2022; Sevanto et al., 2014). Adequate precipitation in spring and autumn contributes significantly to tree growth and is crucial for water transport (Warner et al., 2024; Wu et al., 2025). Carbohydrate reserves accumulated from precipitation in the previous summer and autumn are essential for earlywood formation and radial growth in the following year (Galiano et al., 2011; Wu et al., 2025). Some research pointed out that growing season precipitation and the Palmer Drought Severity Index (PDSI) are the main controls on urban tree growth, with temperature playing a secondary role (Gillner et al., 2014; Rakthai et al., 2020). Furthermore, fast-growing tree species are more susceptible to drought due to their high soil water consumption (Jing et al., 2022; Li et al., 2026; Marchin et al., 2022). Although the growth response to climate varies among regions due to species specific and habitat specific factors, there is a consensus that recent urban warming and drying trends have negative impacts on tree growth. Our results are in line with these findings: tree growth in both urban and suburban areas was significantly influenced by water availability in spring and autumn (**Figure 4**). Therefore, adequate water is a key limiting factor for tree growth in subtropical cities, and urban trees are more vulnerable to water stress.

When reconciling our findings with studies that reported urban warming promotes tree growth, the distinct climatic contexts must be considered. In temperate cities, temperature often limits growth, and warming can extend the growing season and enhance growth. However, in humid subtropical regions such as our study area, water availability is the predominant limiting factor. Rising temperatures intensify evapotranspiration and atmospheric vapor pressure deficit (VPD), increasing plant water demand and inducing stomatal constraints on carbon assimilation. Indeed, VPD has been identified as the most important factor influencing daily growth and growth probability in subtropical urban trees, and increased VPD leads to greater decoupling between carbon source and sink (Zheng et al., 2023). Furthermore, global evidence indicates that atmospheric dryness imposes stronger constraints on vegetation in tropical/subtropical zones than in temperate zones (Yan W. et al., 2024). Therefore, in humid subtropical cities, the negative effects of warming-driven increases in evapotranspiration and VPD likely outweigh any potential benefits of a longer growing season. This explains why, in our study, urban trees did not exhibit growth enhancement but rather showed greater vulnerability to drought and lower resilience compared to their suburban counterparts.

### 4.2 Differential responses of urban and suburban trees to extreme drought

Using tree ring analysis and an ecological resilience quantification framework, this study systematically revealed the differences in response to extreme drought events among four common greening tree species in the urban and suburban areas of Nanchong City (**Figures 5**, **6**). The results support our hypothesis: urban trees generally exhibited lower ecological resilience, particularly during the severe drought of 2011, when their resistance and recovery were significantly lower than those of the same species in suburban areas. This finding is highly consistent with recent global studies (Esperon-Rodriguez et al., 2025c; Nitschke et al., 2017; Yang et al., 2026). For example, a meta-analysis found that 68% of urban trees showed a declining trend in resilience over the past decades (Esperon-Rodriguez et al., 2022). These lines of evidence indicate that the weakening of tree climate adaptability by urban environments is a cross regional phenomenon (Esperon-Rodriguez et al., 2025d).

The resistance patterns provided partial support for H1, with urban trees exhibiting lower Rt than suburban trees in 2006 but comparable Rt in 2011, indicating that the urban–suburban resistance gap is event-dependent. The higher Rt of urban *C. deodara* in 2006 may reflect its inherent drought tolerance, combined with potential benefits from the urban heat island effect, supplemental irrigation, and urban-induced physiological adjustments that enhance drought tolerance traits in some species (Rosenberger et al., 2025; Wu et al., 2025). The disappearance of the urban–suburban Rt difference in 2011 may reflect cumulative stress burden following previous drought exposure, as well as event-specific differences in drought severity. Importantly, despite variable resistance, urban trees consistently exhibited significantly lower recovery and resilience than suburban trees across both events (**Figure 5**), suggesting that the primary vulnerability of urban trees lies in post-drought recovery rather than immediate drought resistance (Yang et al., 2026). This is consistent with the trade-off between resistance and recovery documented in other urban forests and with the view that urban environments impose persistent physiological stress that compromises trees’ ability to replenish carbon reserves and restore hydraulic function after drought (Tan et al., 2025).

Notably, resilience performance varied markedly among tree species, highlighting the central importance of functional type classification in urban greening planning. *Cinnamomum camphora*, a native dominant evergreen broad leaved species, maintained relatively high resilience in urban areas during the extreme drought of 2011, with recovery superior to that of other species. This is closely related to its physiological traits, including deep roots, high water use efficiency (WUE), and well-developed leaf cuticles (Wang et al., 2025; Zhang et al., 2022), indicating that it possesses strong climate buffering capacity and is a preferred tree species for subtropical urban greening. This result echoes the “native species resilience advantage” reported by Zhang et al. (2022) in *Pinus tabulaeformis* in Beijing (Wang et al., 2020). In contrast, for poplar (ring porous), the resilience and recovery of urban trees were significantly lower than those of suburban trees during the extreme droughts of 2006 and 2011, consistent with its anatomical characteristics of large earlywood vessels and weak embolism resistance (Choat et al., 2012; Fickle et al., 2025; Rimer et al., 2026; Xia and Zhang, 2026). A recent study in northern Chinese cities also indicated that ring porous species are more prone to hydraulic failure under combined heat and drought stress (Lv et al., 2025). Of concern, the “illusory prosperity” trap of fast-growing urban trees with low resilience (Franceschi et al., 2023), is also observed in the present study: urban poplars had higher mean annual growth increments than suburban poplars but lower resilience. Therefore, in subtropical cities with frequent heatwaves and droughts, the use of highly sensitive fast-growing species such as poplar should be carefully considered, especially avoiding large scale planting in high-density built-up areas.

### 4.3 Research limitations

It should be acknowledged that the urban trees sampled in this study, particularly those in parks and along streets, are subject to routine management practices, including supplemental irrigation during hot and dry seasons, as well as pruning and fertilization, as part of the city’s regular urban greening maintenance programs. This management practice is a common feature of urban environments and may artificially alleviate water stress to some extent, potentially serving as a confounding factor that could lead to an overestimation of urban trees’ intrinsic drought recovery capacity. However, despite this supplemental watering, our results still revealed significantly lower recovery and resilience in urban trees compared to their suburban counterparts (**Figure 5**), indicating that the combined stresses of the urban heat island effect, soil compaction, and limited water infiltration impose a persistent physiological burden that cannot be fully offset by routine irrigation (Madrigal-González et al., 2024). Furthermore, the typically shallow and episodic nature of urban irrigation may not adequately mimic natural soil moisture regimes. Recent studies have shown that weekly irrigation fails to prevent drought stress for trees with limited root space (Rosenberger et al., 2025), and that irrigated urban trees may develop very shallow root systems, making them more susceptible to drought (Bijoor et al., 2012). Therefore, the observed urban–suburban differences in resilience likely represent a conservative estimate of the true ecological costs of urbanization on tree health.

The observed urban–suburban differences are influenced not only by water availability but also by multiple interacting stressors and management practices (Esperon-Rodriguez et al., 2025b; Zhao et al., 2014). Urban trees are subject to soil compaction, restricted rooting space, and air pollution, which collectively impair root development, reduce water infiltration, and compromise physiological functions (Madrigal-González et al., 2024; Rosenberger et al., 2025; Tan et al., 2025; Yang et al., 2026). Compacted urban soils, with bulk densities often exceeding 1.56 g/cm³, restrict fine root growth and water infiltration, while limited rooting volume in narrow tree pits exacerbates water stress (Rosenberger et al., 2025). Management practices, including irrigation, fertilization, and pruning, can partially alleviate these stresses; however, routine irrigation does not necessarily eliminate drought stress, particularly when root space is limited, and may promote shallow root systems that reduce long-term resilience (Rochner et al., 2025; Rosenberger et al., 2025). Therefore, the higher resilience observed in urban *C. camphora* during the 2011 drought should be interpreted with caution. While this species possesses intrinsic drought adaptive traits, including a conservative hydraulic strategy and high embolism resistance, the contribution of park irrigation and other management inputs cannot be ruled out (Sweet et al., 2025). The superior performance of urban *C. camphora* likely reflects a synergistic effect of inherent physiological tolerance and site-specific management practices, rather than an intrinsic resilience advantage independent of human intervention (Wang et al., 2025). Furthermore, the increased resistance in urban trees may still be insufficient to fully offset the pressures of urbanization on tree growth and resilience. It is also noteworthy that many urban greening tree species originate from different climatic zones, and potential mismatches between future climate adaptability and urban environments may further exacerbate stress risks for urban trees (Esperon-Rodriguez et al., 2021; Esperon-Rodriguez et al., 2022). Consequently, our conclusion that native species may have advantages in urban environments should be considered preliminary and context dependent. Future studies should explicitly account for management intensity, soil physical properties, air quality, and climatic suitability to disentangle the relative contributions of species traits and anthropogenic factors to urban tree resilience.

Several limitations of this study should be acknowledged. First, our resilience analyses are based on only two extreme drought events (2006 and 2011). This limited number of events restricts our ability to capture the full spectrum of drought variability, and the observed responses may be event-specific (Schwarz et al., 2020). Second, the reference periods for the two drought events partially overlap, which may confound the recovery signal of the 2011 event with the legacy effects of the 2006 drought (Lloret et al., 2011). Third, our findings are derived from a single subtropical city (Nanchong); caution is therefore warranted when extrapolating these results to other climatic regions or cities with different urbanization histories and management practices (Esperon-Rodriguez et al., 2022). Fourth, while we discussed the potential influences of urban management (e.g., irrigation) and other stressors (e.g., soil compaction, air pollution), these factors were not explicitly controlled for in our study design (Esperon-Rodriguez et al., 2025a). The observed urban–suburban differences should thus be interpreted as the net outcome of multiple interacting environmental and anthropogenic factors, rather than the effect of any single driver. Future research with multi-city comparisons, longer drought records, and quantitative management data is needed to further validate and extend our findings.

## 5 Conclusion

This study reveals the growth responses of trees to climate change in both urban and suburban areas of Nanchong, a Chinese subtropical city. The results show that trees in both urban and suburban areas are significantly affected by urban climate change: water deficit is the main limiting factor for tree growth in both areas, with a stronger impact on urban trees, especially for species with higher water requirements such as *Metasequoia glyptostroboides*. Under extreme drought conditions, urban trees exhibit lower recovery and resilience compared to suburban trees. However, for the native urban species *Cinnamomum camphora*, both recovery and resilience are substantially higher than those in suburban areas. Over recent decades, the four tree species in the urban area have shown a negative or stagnant growth trend, likely due to water limitation induced by rising urban temperatures. In contrast, the growth rate of suburban trees is much faster than that of urban trees. As urban expansion continues, urban temperatures will keep rising, imposing higher thermal stress on urban trees and adversely affecting their growth. Therefore, urban managers could mitigate the impact of drought stress on urban trees by improving soil water availability through irrigation or planting native tree species. Future research should integrate remote sensing inversion, process-based models, and tree ring data to establish a comprehensive assessment framework of urban forests, thereby providing solid scientific support for building climate adaptive and ecologically resilient cities.

## Data Availability

The original contributions presented in the study are included in the article/**Supplementary Material**. Further inquiries can be directed to the corresponding author.
