# Supplementary material for: Urban–suburban differences in the response of typical greening tree species to extreme drought in a subtropical city
Source: Front Plant Sci. 2026 Aug 10;17:1900663. doi: 10.3389/fpls.2026.1900663 (PMC13500766; doi:10.3389/fpls.2026.1900663)
Supplement: Supplementary file 1 [file DataSheet1.docx]

Supplementary Material

# 1 Supplementary Figures and Tables

## 1.1 Supplementary Figures

**FIGURE S1.** Temporal variations in climatic variables of urban and suburban areas in Nanchong from the Gaoping and Xichong weather stations during 1995-2022.

**FIGURE S2.** Detrended tree-ring width index of urban and suburban trees.

**FIGURE S3.** The correlation diagram shows the relationship (Pearson correlation coefficient) between climate variables and eight tree-ring width chronologies of in suburban and urban areas during the period 1995–2022. (a) mean temperature , (b) total precipitation and (c) SPEI.

**FIGURE S4.** Temporal trends of basal area increment (BAI) for four tree species of urban and suburban areas in Nanchong during the period 1995–2022. The symbol "β" represents the slope of BAI (cm² yr⁻¹). The linear regression results of each period are attached.

## 1.2 Supplementary Tables

# Table S1. Information on sampling sites in the urban and suburban areas of Nanchong, China. DBH, Diameter at breast height.

| Tree species | Location | Site name | Altitude /m | Longitude /°E | Latitude /°N | DBH  /cm | Tree height /m |
| --- | --- | --- | --- | --- | --- | --- | --- |
| *Cinnamomum camphora* | Urban | XHHFs | 238 | 106.088 | 30.800 | 51.4±7.9 | 18.2±2.1 |
|  | Suburban | NCGHs | 266 | 106.064 | 30.817 | 41.2±6.5 | 9.0±0.7 |
| *Metasequoia glyptostroboides* | Urban | XHHFa | 265 | 106.062 | 30.794 | 38.8±4.7 | 11.3±2.2 |
|  | Suburban | NCXSa | 240 | 106.062 | 30.820 | 18.6±4.1 | 5.3±1.5 |
| *Cedrus deodara* | Urban | NCCYx | 260 | 106.070 | 30.787 | 36.9±5.9 | 11.5±2.2 |
|  | Suburban | NCXHx | 232 | 106.068 | 30.820 | 41.0±7.1 | 8.0±3.2 |
| *Populus ‘Zhonghua Hongye’* | Urban | HFXHy | 237 | 106.140 | 30.770 | 43.7±8.2 | 11.9±4.7 |
|  | Suburban | GPDSy | 272 | 106.060 | 30.820 | 60.2±9.0 | 15.8±1.8 |

# Table S2. Statistics of tree-ring width chronologies of urban and suburban areas in Nanchong, China. Rbar, the mean inter-series correlation; MS, mean sensitivity; AC1, 1st order autocorrelation; SNR, signal-to-noise ratio; EPS, expressed population signal.

| Tree species | *Metasequoia glyptostroboides* | | *Cinnamomum camphora* | | *Cedrus deodara* | | | *Populus 'Zhonghua Hongye'* | |
| --- | --- | --- | --- | --- | --- | --- | --- | --- | --- |
| Location | Suburban | Urban | Suburban | Urban | | Suburban | Urban | Suburban | Urban |
| Site name | XHHFs | NCGHs | XHHFa | NCXSa | | NCCYx | NCXHx | HFXHy | GPDSy |
| Cores/ Trees | 37/37 | 23/23 | 27/27 | 13/13 | | 31/31 | 11/11 | 24/24 | 13/13 |
| Period | 2005-2022 | 1995-2022 | 1991-2020 | 1976-2020 | | 2000-2021 | 2002-2021 | 1989-2022 | 1996-2022 |
| Stand mean age | 13±2.18 | 17±4.48 | 24±3.83 | 41±6.22 | | 20±2.59 | 19±2.62 | 26±6.22 | 20±3.98 |
| MS | 0.365 | 0.475 | 0.267 | 0.174 | | 0.218 | 0.27 | 0.162 | 0.212 |
| AC1 | 0.265 | 0.173 | 0.392 | 0.324 | | 0.451 | 0.425 | 0.289 | 0.342 |
| Rbar | 0.483 | 0.642 | 0.606 | 0.564 | | 0.261 | 0.371 | 0.384 | 0.454 |
| SNR | 34.567 | 41.246 | 41.528 | 16.817 | | 10.949 | 6.488 | 14.961 | 10.810 |
| EPS | 0.972 | 0.976 | 0.976 | 0.944 | | 0.916 | 0.866 | 0.937 | 0.915 |

# Table S3. Statistic parameters of multiple regression models. For model construction, we began with a full model and subsequently removed variables that exerted the greatest influence on the AICc. Furthermore, based on the optimal model, we performed model averaging (ΔAICc < 2) to determine the parameter coefficients for the final predictor set of tree growth. “*”, “**”, “***” represents significant relationship at level of *p* < 0.05, *p* < 0.01, *p* < 0.001. Abbreviations: T_Spr = spring mean temperature; T_Sum = summer mean temperature; T_Aut = autumn mean temperature; SPEI_pAut = previous autumn standardized precipitation-evapotranspiration index; SPEI_Spr = spring standardized precipitation-evapotranspiration index; SPEI_Aut = autumn standardized precipitation-evapotranspiration index; Pre_pAut = previous autumn total precipitation; Pre_Aut = autumn total precipitation.

|  | Variable | Estimate | Standard Error | t value | p-value |
| --- | --- | --- | --- | --- | --- |
| Urban | Intercept | -0.2429 | 0.1106 | -2.196 | 0.036868* |
|  | Pre_pAut | -0.9632 | 0.222 | -4.338 | 0.00018*** |
|  | SPEI_Aut | 0.7335 | 0.1561 | 4.698 | 0.0000686*** |
|  | SPEI_pAut | 0.9694 | 0.1796 | 5.398 | 0.0000105*** |
|  | SPEI_Spr | -0.3201 | 0.1252 | -2.557 | 0.016509* |
|  | T_Aut | -0.5977 | 0.147 | -4.066 | 0.000372*** |
|  | T_Spr | 0.3545 | 0.1534 | 2.311 | 0.028712* |
| Suburban | Intercept | -0.09458 | 0.11566 | -0.818 | 0.420405 |
|  | Pre_Aut | 0.5744 | 0.14769 | 3.889 | 0.000565*** |
|  | Pre_pAut | 0.29397 | 0.11675 | 2.518 | 0.0178* |
|  | SPEI_Spr | -0.53471 | 0.1349 | -3.964 | 0.000463*** |
|  | T_Sum | 0.47933 | 0.14473 | 3.312 | 0.002562** |
